# Patient-reported outcomes during first-line palliative systemic therapy alternated with pressurized intraperitoneal aerosol chemotherapy for unresectable colorectal peritoneal metastases: a single-arm phase II trial (CRC-PIPAC-II)

**DOI:** 10.1007/s00464-024-11185-z

**Published:** 2024-09-16

**Authors:** Vincent C. J. van de Vlasakker, Paulien Rauwerdink, Koen. P. B. Rovers, Emma C. Wassenaar, Geert-Jan Creemers, Maartje Los, Jacobus . W. A. Burger, Simon W. Nienhuijs, Onno Kranenburg, Marinus J. Wiezer, Robin J. Lurvink, Djamila Boerma, Ignace H. J. T. de Hingh

**Affiliations:** 1 Department of Surgery, Catharina Hospital, Catharina Cancer Institute, PO Box 1350, 5602 Eindhoven, ZA The Netherlands; 2 Department of Surgery, St. Antonius Hospital, Nieuwegein, The Netherlands; 3 Department of Medical Oncology, Catharina Hospital, Eindhoven, The Netherlands; 4 Department of Medical Oncology, St. Antonius Hospital, Nieuwegein, The Netherlands; 5 Lab Translational Oncology, Division Imaging and Cancer, University Medical Center Utrecht, Utrecht, The Netherlands; 6 Research and Development, Netherlands Comprehensive Cancer Organisation, Utrecht, The Netherlands; 7 GROW- School for Oncology and Development Biology, Maastricht University, Maastricht, The Netherlands

**Keywords:** PIPAC, Patient reported outcomes, Linear mixed modeling, Quality of life, Colorectal peritoneal metastases

## Abstract

**Background:**

The CRC-PIPAC-II study prospectively assessed bidirectional therapy (BT) consisting of first-line palliative systemic therapy and electrostatic precipitation oxaliplatin-based pressurized intraperitoneal aerosol chemotherapy (ePIPAC-OX) in patients with unresectable colorectal peritoneal metastases (CPM). This study describes the exploration of patient-reported outcomes (PROs).

**Methods:**

In this phase II trial, 20 patients with isolated CPM were treated with up to three cycles of BT, each cycle consisting of two to three courses of systemic therapy, followed by ePIPAC-OX (92 mg/m^2^). Patients were asked to complete the EuroQoL EQ-5D-5L, EORTC QLQ-C30, and EORTC QLQ-CR29 questionnaires at baseline, during the first cycle of BT, and one and four weeks after each consecutive BT cycle. PRO scores were calculated and compared between baseline and each subsequent time point using linear-mixed modeling (LMM). PROs were categorized into symptom scales and function scales. Symptom scales ranged from 0 to 100, with 100 representing the maximum symptom load. Function scales ranged from 0 to 100, with 100 representing optimal functioning.

**Results:**

Twenty patients underwent a total of 52 cycles of bidirectional therapy. Most PROs (29 of 37, 78%) were not significantly affected during trial treatment. In total, only eight PROs (22%) were significantly affected during trial treatment: Six PROs (index value, global health status, emotional functioning, C30, appetite, and insomnia) showed transient improvement at different time points. Two PROs transiently deteriorated: pain initially improved during the first BT cycle [− 16, *p* < 0.001] yet worsened temporarily one week after the first two BT cycles (+ 20, *p* < 0.001; + 17, *p* = 0.004; respectively). Abdominal pain worsened temporarily one week after the first BT cycle (+ 16, *p* = 0.004), before improving again four weeks after treatment ended (− 10, *p* = 0.004). All significant effects on Pros were clinically significant and all deteriorations in PROs were of temporary nature.

**Discussion:**

Patients undergoing BT for unresectable CPM had significant, but reversible alterations in several PROs. Most affected PROs concerned improvements and only two PROs showed deteriorations. Both deteriorated PROs returned to baseline after trial treatment and were of a temporary nature. These outcomes help to design future studies on the role of ePIPAC in the palliative setting.

**Supplementary Information:**

The online version contains supplementary material available at 10.1007/s00464-024-11185-z.

Patients diagnosed with peritoneal metastases from colorectal origin (CPM) are characterized by a poor prognosis. Only a small minority (10–15%) is eligible for treatment with curative intent, while the majority of CPM patients (60–65%) will undergo palliative systemic treatment [1]. This is thought to be less effective in CPM as compared to other metastatic sites from colorectal origin [1–4]. One of the workarounds to circumvent this relative inefficacy is to administer chemotherapy directly into the peritoneal cavity [4]. However, the use of intraperitoneal chemotherapy is limited by poor tumor penetration, inhomogeneous intraperitoneal drug distribution and local toxicity [5, 6].

Pressurized intraperitoneal aerosol chemotherapy (PIPAC) was developed to overcome these limitations [7–9]. PIPAC is a laparoscopic method for the intraperitoneal administration of chemotherapy as a pressurized aerosol and has been implemented in a substantial number of centers worldwide [10]. Generally, CPM patients are treated with oxaliplatin-based PIPAC (92 mg/m^2^, PIPAC-OX) every 6–8 weeks. Previously, we investigated the safety of repetitive electrostatic PIPAC-OX (ePIPAC-OX) as a palliative monotherapy in CPM patients in a phase II study (CRC-PIPAC-I) [11]. Other study outcomes involved the exploration of patient-reported outcomes (PROs). PROs are important outcome measures assessing health-related quality of life (QOL) and are one of the principal outcomes to palliative care interventions, yet currently understudied in the setting of (e)PIPAC [14]. Regarding PROs assessed in the CRC-PIPAC study, it was concluded that patients receiving ePIPAC-OX monotherapy had reversible worsening of several clinically relevant PROs, the foremost of which was abdominal pain [11].

In order to improve the dismal prognosis of CPM patients, repetitive (e)PIPAC-OX can also be alternated with palliative systemic therapy to potentially intensify anti-tumor activity, henceforth referred to as bidirectional therapy [12, 13]. The impact of this intensified treatment on PROs is currently unclear. We recently performed the CRC-PIPAC-II trial, investigating the safety and efficacy of combining ePIPAC-OX with palliative systemic therapy in 20 patients with CPM [14]. The present manuscript aimed to gain more insight into the impact of this treatment on PROs of patients with unresectable CPM during treatment.

## Methods

The CRC-PIPAC-II was a single-arm, phase II trial conducted in two tertiary referral centers for CPM (International Clinical Trials Registry Platform: NL8303). The Central Medical Ethics Committee (MEC-U, Nieuwegein, the Netherlands, R19.087) and the institutional review boards of both participating institutions approved the study. The study protocol was published previously; therefore, the present manuscript only provides a brief summary [14].

### Patient population

Patients were eligible for participation if they were at least 18 years old and presented with a World Health Organization (WHO) performance score of 0–1, histopathological confirmation of colorectal or appendiceal peritoneal metastases that were deemed unresectable through surgical and/or radiological assessment, without symptoms of gastro-intestinal obstruction, with adequate organ functions, no contraindications for chemotherapy or laparoscopy, no prior treatment with palliative chemotherapy for CRC, no treatment with systemic chemotherapy within 6 months prior to inclusion, and no prior PIPAC procedures. Written informed consent was obtained from all patients.

### Bidirectional therapy

Patients received up to three cycles of bidirectional therapy. Each cycle consisted of 6 weeks of first-line palliative systemic therapy (e.g., two to three courses of systemic therapy) followed by one ePIPAC-OX procedure within 1 to 4 weeks after completion of systemic therapy. Trial treatment ended after the third ePIPAC-OX procedure. Trial treatment could be terminated prematurely due to disease progression, toxicity, at the patient’s request, or at the physician’s discretion.

### First-line palliative systemic therapy

The treating medical oncologist determined which of the following regimens was used:

1. Two three-weekly cycles of CAPOX-bevacizumab (intravenous oxaliplatin (130 mg/m2 body surface area [BSA]) on day one, oral capecitabine (1000 mg/m2 BSA) twice daily on days one–fourteen, and intravenous bevacizumab (7.5 mg/kg body weight) on day one);
2. Three two-weekly cycles of FOLFOX-bevacizumab (intravenous oxaliplatin (85 mg/m^2^ BSA) on day one, intravenous leucovorin (400 mg/m^2^ BSA) on day one, intravenous bolus/continuous 5-fluorouracil (400/2400 mg/m^2^ BSA) on days one–two, and intravenous bevacizumab (5 mg/kg body weight) on day one);
3. Three two-weekly cycles of FOLFIRI-bevacizumab (intravenous irinotecan (180 mg/m^2^ BSA) on day one, intravenous leucovorin (400 mg/m^2^ BSA) on day one, 5-fluorouracil (400/2400 mg/m^2^ BSA) on day one, and intravenous bevacizumab (5 mg/kg body weight) on day one).
4. Three two-weekly cycles of FOLFOXIRI-bevacizumab (intravenous oxaliplatin (85 mg/m^2^ BSA) on day one, intravenous irinotecan (165 mg/m^2^ BSA) on day one, intravenous leucovorin (400 mg/m^2^ BSA) on day one, intravenous continuous 5-fluorouracil (2400 mg/m^2^ BSA) on days one–two, and intravenous bevacizumab (5 mg/kg body weight) on day 1).

Dose reductions and switches between allowed regimens and management of toxicity were left to the discretion of the treating medical oncologist.

### ePIPAC-OX

Within one to four weeks after completion of each cycle of first-line systemic therapy, patients were treated with ePIPAC under general anesthesia with an initial intravenous bolus of leucovorin (20 mg/m^2^ in 10 min) and 5-fluorouracil (400 mg/m^2^ in 15 min), followed by intraperitoneal administration of oxaliplatin (92 mg/m^2^ BSA). Oxaliplatin was prepared in a total volume of 150-mL dextrose solution and injected through the nebulizer (CapnoPen, Capnomed GmbH, Villingendorf, Germany) in 5 min, after which the Ultravision generator (Ultravision, Alesi Surgical, Cardiff, UK) administered electrostatic precipitation to the aerosol. The electrostatic field and the 12-mm Hg capnoperitoneum were then maintained at 37 °C for another 25 min. For a complete description of the ePIPAC procedure, we refer to the study protocol [14].

### PROs

Patients were asked to complete three questionnaires (EuroQoL EQ-5D-5L [15], EORTC QLQ-C30 [16], and EORTC QLQ-CR29 [17]) at baseline, one week before the first PIPAC procedure, and one and four weeks after each PIPAC procedure. As the aim of the study was to assess PROs during bidirectional therapy, patients were no longer asked to complete questionnaires after trial discontinuation or after having completed the entire trial treatment. All PROs collected up to the point of discontinuation were included in the analyses regardless of the reason for discontinuation.

Scores for each PRO category were calculated following the manuals of the EuroQol and EORTC [18–20]. The index value of the EuroQol EQ-5D-5L ranges from − 0.329 to 1.00, as determined in the Dutch value set [21]. All other EORTC scores range from 0 to 100, where higher scores represent better functioning in the function scales (Table 1) and lower scores represent worse symptom load in the symptom scales (Table 2), respectively.

**Table 1:** Patient-reported outcomes (PROs) per questionnaire

Questionnaire	Function scales*	Symptom scales**
EQ-5D-5L	visual analog scale, index value	–
EORTC-QLQ-C30	global health status, physical functioning, role functioning, emotional functioning, cognitive functioning, social functioning, C30 summary score	Fatigue, nausea/vomiting, pain, dyspnea, insomnia, appetite loss, constipation, diarrhea, financial difficulties
EORTC-QLQ-CR29	anxiety, weight, body image, sexual interest (males and females)***	urinary frequency, urinary incontinence, dysuria, abdominal pain, buttock pain, bloating, blood/mucus in stool, dry mouth, hair loss, taste, flatulence, fecal incontinence, sore skin, stool frequency, embarrassment, stoma care problems, impotence (males)***, dyspareunia (females)***

*lower scores indicate worse functioning

**higher scores indicate worse symptoms

***excluded from mixed-linear-model analysis due to too many missing values

**Table 2:** Baseline characteristics of patients treated with bidirectional therapy

Characteristics	Patients (*n* = 20)
Age (years), median (range)	57.5 (41–70)
*Sex*	
Female	10 (50)
Male	10 (50)
*WHO performance status*	
0	14 (70)
1	6 (30)
*Primary tumor location*	
Right colon	9 (45)
Left colon	2 (10)
Appendix	9 (45)
*Histology primary tumor*	
Adenocarcinoma	5 (25)
Adenocarcinoma with signet ring cells^a^	3 (15)
Mucinous adenocarcinoma	7 (35)
Mucinous adenocarcinoma with signet ring cells^a^	1 (5)
Goblet cell adenocarcinoma	2 (10)
Low-grade appendiceal mucinous neoplasm^b^	1 (5)
Unknown^c^	1 (5)
*Primary tumor resection status*	
Resected	6 (30)
In situ	14 (70)
*Colostomy, ileostomy*	3 (15)
*Chemotherapy naïve, no prior systemic therapy*	20 (100%)
*Onset of peritoneal metastases*	
Synchronous	16 (80)
Metachronous	4 (20)
*Radiological PCI at enrollment, median (range)*	27 (15–38)

Data are expressed as *n* (%) unless otherwise specified

*WHO* world health organization, *PCI* peritoneal cancer index

^a^Primary tumor comprised of less than 50% signet ring cell components

^b, c^Pre-enrollment peritoneal biopsies classified as colorectal mucinous adenocarcinoma, restaged during trial as low-grade pseudomyxoma peritonei (PMP) originating from a low-grade appendiceal mucinous neoplasm^b^, and high-grade PMP originating from appendiceal tumor of unknown histologic grade (appendix in situ)^c^, respectively

^d^Mutation status could not be evaluated due to insufficient patient material with vital tumor cells

### Statistical analysis

Due to the exploratory nature of the study, no a priori hypotheses were formulated and as such, all analyses were performed in a two-sided fashion. To correct for multiple testing, a Bonferroni correction per item was performed, resulting in a *p* value of < 0.0071 (0.05/7 = 0.0071 [7 comparisons to baseline]) to indicate statistically significant difference. All analyses were performed using IBM SPSS Statistics (version 25.0, Armonk, NY, United States). Categorical baseline characteristics were presented as *n* (%). For all PRO items, score changes between baseline and all seven subsequent time points were presented as mean differences (MD) and compared using linear-mixed modeling (LMM). For LMM a maximum likelihood estimation and an unstructured covariance matrix were used. For all PRO categories with statistically significant differences between baseline and subsequent time points, the Cohen’s D (CD) was calculated to determine the clinical relevance of the statistical difference (i.e., CD > 0.5 being considered clinically relevant) [22]. Furthermore, clinical thresholds were used to determine the extent of the deteriorations and improvements (i.e., minor [CD 0.2–0.5], moderate [CD 0.5–0.8], or major [CD > 0.8]) for the EORTC QLQ-C30 and EORTC QLQ-CR29 [23, 24]. For the EQ5D5, the CD was used to determine whether changes exceeded the threshold for minimally important difference (i.e., CD > 0.5) [25].

## Results

**Fig. 1:**
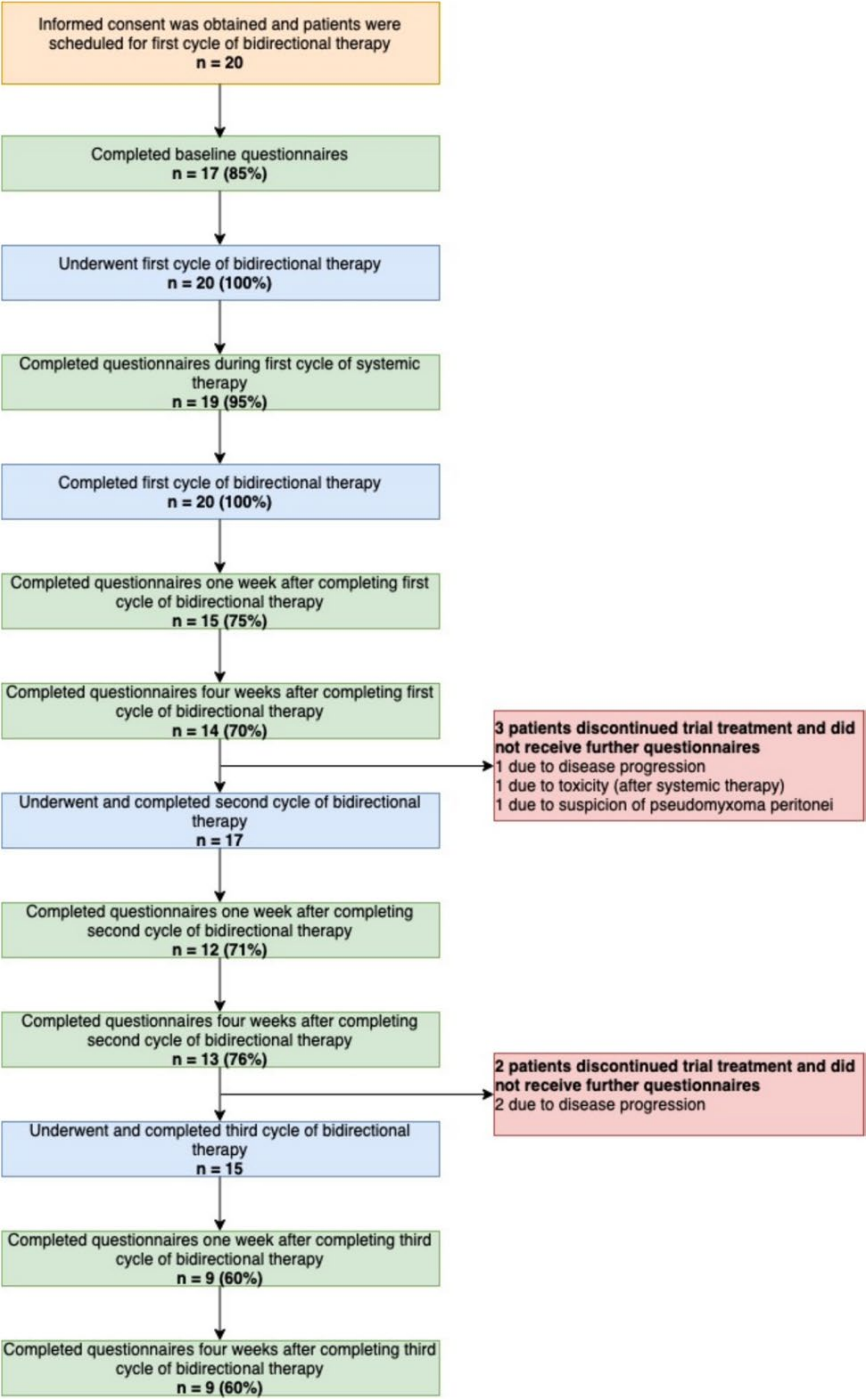
Study flowchart

Between February 2020 and August 2022, 20 patients were included in the study and were treated with a total of 52 cycles of bidirectional therapy. Figure 1 represents the flow of patient inclusion, treatment course, and questionnaire response rate. Baseline characteristics are presented in Table 2**.**

Response rates to the questionnaires varied between 95% (19/20, before the first PIPAC procedure) and 60% (9/15, four weeks after the last PIPAC procedure). (Fig. 1).

As fewer than 30% of both male and female patients completed PROs questions regarding sexual interest and sexual function (EORTC-QLQ-CR29), these PRO categories were excluded from the current analysis.

The mean scores of eight different PRO categories changed significantly during the trial treatment and are discussed below, whereas the mean scores of the remaining 29 PRO categories did not change significantly (Table 3). The LMM analyses of all 37 PRO categories are presented in Supplementary Table 1.

**Table 3:** Mean scores with standard deviations of PROs at each time point

PRO	Baseline	1 week before PIPAC-1	1 week after PIPAC-1	4 weeks after PIPAC-1	1 week after PIPAC-2	4 weeks after PIPAC-2	1 week after PIPAC-3	4 weeks after PIPAC-3
*EQ-5D-5L*								
Visual analog scale	63 ± 23	68 ± 22	61 ± 28	67 ± 23	62 ± 14	67 ± 14	72 ± 17	68 ± 14
Index value	0,74 ± 0,15	0,86 ± 0,08	0,70 ± 0,21	0,80 ± 0,12	0,76 ± 0,13	0,78 ± 0,12	0,91 ± 0,21	0,88 ± 0,13
*EORTC QLQ-C30*						4		
Global health status	65 ± 23	78 ± 15	60 ± 28	67 ± 13	59 ± 20	65 ± 19	54 ± 22	77 ± 16
Physical functioning	80 ± 23	86 ± 21	69 ± 25	84 ± 14	76 ± 19	80 ± 13	69 ± 25	83 ± 17
Role functioning	62 ± 32	78 ± 25	53 ± 28	72 ± 20	47 ± 28	63 ± 23	69 ± 26	63 ± 27
Emotional functioning	70 ± 23	84 ± 13	77 ± 17	77 ± 24	80 ± 15	77 ± 17	83 ± 15	90 ± 12
Cognitive functioning	83 ± 24	90 ± 17	84 ± 21	92 ± 14	89 ± 21	79 ± 26	78 ± 26	82 ± 22
Social functioning	73 ± 33	90 ± 15	74 ± 21	81 ± 22	71 ± 20	86 ± 17	69 ± 25	76 ± 22
Fatigue	36 ± 24	28 ± 19	49 ± 30	42 ± 26	56 ± 33	35 ± 24	49 ± 28	28 ± 25
Nausea/vomiting	13 ± 25	1 ± 4	25 ± 37	14 ± 17	23 ± 21	9 ± 22	35 ± 28	8 ± 12
Pain	23 ± 23	7 ± 12	43 ± 23	18 ± 21	40 ± 29	31 ± 27	48 ± 30	31 ± 26
Dyspnea	5 ± 12	7 ± 18	9 ± 21	5 ± 19	11 ± 30	9 ± 21	10 ± 24	7 ± 15
Insomnia	40 ± 34	23 ± 22	33 ± 20	24 ± 22	11 ± 22	30 ± 23	15 ± 29	12 ± 18
Appetite loss	33 ± 31	9 ± 19	41 ± 43	27 ± 30	16 ± 17	18 ± 26	8 ± 35	15 ± 39
Constipation	14 ± 21	7 ± 18	18 ± 25	12 ± 21	17 ± 21	15 ± 22	11 ± 24	*
Diarrhea	14 ± 24	13 ± 19	33 ± 38	17 ± 28	14 ± 17	25 ± 34	19 ± 29	15 ± 22
Financial difficulties	6 ± 24	5 ± 17	4 ± 17	7 ± 19	6 ± 19	5 ± 18	7 ± 22	4 ± 11
C30 summary score	74 ± 16	86 ± 9	67 ± 15	75 ± 13	71 ± 13	73 ± 14	67 ± 15	72 ± 15
*EORTC QLQ-CR29*								
Urinary frequency	32 ± 19	24 ± 26	27 ± 16	20 ± 19	26 ± 19	24 ± 24	25 ± 28	24 ± 19
Urinary incontinence	3 ± 15	0 ± 0	2 ± 0	1 ± 0	0 ± 0	3 ± 9	4 ± 11	7 ± 22
Dysuria	3 ± 10	3 ± 11	2 ± 9	2 ± 9	3 ± 19	2 ± 9	0 ± 0	2 ± 11
Abdominal pain	30 ± 26	16 ± 17	46 ± 25	24 ± 35	39 ± 38	27 ± 30	36 ± 26	20 ± 25
Buttock pain	2 ± 2	0 ± 2	4 ± 2	0 ± 2	0 ± 2	3 ± 2	4 ± 3	4 ± 3
Bloating	25 ± 24	13 ± 20	37 ± 34	26 ± 30	38 ± 40	30 ± 30	48 ± 33	24 ± 28
Blood/mucus in stool	0 ± 6	3 ± 11	4 ± 10	0 ± 15	4 ± 0	3 ± 10	6 ± 7	0 ± 0
Dry mouth	20 ± 23	14 ± 23	16 ± 21	18 ± 22	25 ± 35	21 ± 32	33 ± 37	26 ± 28
Hair loss	12 ± 20	12 ± 28	20 ± 37	24 ± 33	22 ± 30	26 ± 31	11 ± 17	11 ± 17
Taste	18 ± 28	5 ± 12	25 ± 38	11 ± 21	17 ± 27	22 ± 32	25 ± 24	18 ± 28
Flatulence	18 ± 25	22 ± 27	20 ± 27	21 ± 33	25 ± 33	23 ± 32	20 ± 35	20 ± 31
Fecal incontinence	7 ± 14	7 ± 18	4 ± 12	4 ± 12	6 ± 13	8 ± 15	11 ± 24	4 ± 11
Sore skin	7 ± 14	11 ± 22	11 ± 25	6 ± 20	9 ± 22	17 ± 33	10 ± 17	5 ± 15
Stool frequency	19 ± 22	12 ± 19	24 ± 21	10 ± 15	15 ± 22	12 ± 22	12 ± 22	5 ± 11
Embarrassment	10 ± 19	12 ± 20	22 ± 29	8 ± 20	15 ± 27	17 ± 29	15 ± 29	10 ± 24
Stoma care problems	11 ± 19	0 ± 0	0 ± 0	0 ± 0	0 ± 0	0 ± 0	*	*
Anxiety	42 ± 18	55 ± 29	51 ± 21	46 ± 21	32 ± 21	49 ± 17	42 ± 18	42 ± 24
Weight	18 ± 30	23 ± 27	29 ± 35	21 ± 25	11 ± 16	13 ± 17	19 ± 24	15 ± 24
Body image	21 ± 23	15 ± 27	17 ± 20	15 ± 21	26 ± 32	16 ± 26	18 ± 21	22 ± 28

*no data available

### Affected function scales

**Fig. 2:**
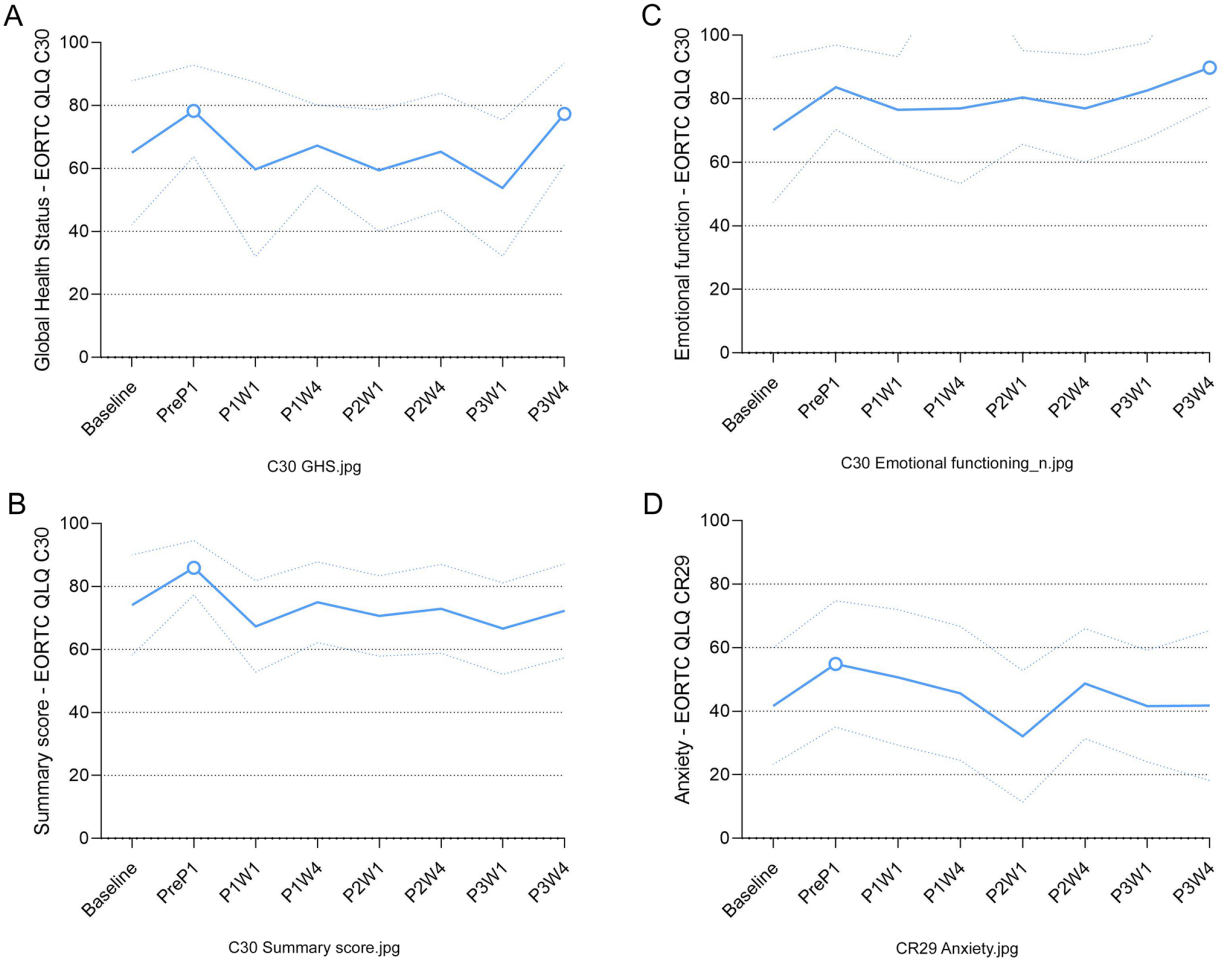
**A**–**D** Function scales with a statistically significant mean difference, indicating statistical difference. PreP1 before first PIPAC procedure; P1W1 one week after first PIPAC procedure; P1W4 four weeks after first PIPAC procedure; P2W1 one week after second PIPAC procedure; P2W4 four weeks after second PIPAC procedure; P3W1 one week after third PIPAC procedure; and P3W4 four weeks after third PIPAC procedure

*Index value*: Compared to baseline, the index value improved after the first cycle of systemic therapy measured one week before the first ePIPAC procedure (MD + 0.12 [95% confidence interval {CI}: 0.05–0.19], *p* = 0.001, CD = 1.02, exceeding the threshold for minimally important difference). Index values did not deviate from baseline at subsequent time points (Fig. 2a).

*Global health status*: Compared to baseline, the global health status improved after the first cycle of systemic therapy measured one week before the first ePIPAC procedure (MD + 13 [95% CI: 5–13], *p* < 0.001, CD = 0.69, moderate improvement). Thereafter, global health status returned to baseline and improved again four weeks after the last ePIPAC procedure (MD + 12 [95% CI: 6–18], *p* < 0.001, CD = 0.62, indicating a moderate improvement) (Fig. 2b).

*Emotional functioning*: Compared to baseline, emotional functioning improved four weeks after the last ePIPAC procedure (MD + 20 [95% CI: 11–29], *p* < 0.001, CD = 1.07, major improvement) (Fig. 2c).

*C30 summary score*: Compared to baseline, the C30 summary score (i.e., the mean score of the 13 domains of the C30 questionnaire) improved after the first cycle of systemic therapy measured one week before the first ePIPAC procedure (MD + 12 [95% CI: 6–18], *p* < 0.001, CD = 0.93, indicating a major improvement). Hereafter, it returned to baseline and did not deviate from baseline at subsequent time points (Fig. 2d).

### Affected symptom scales

**Fig. 3:**
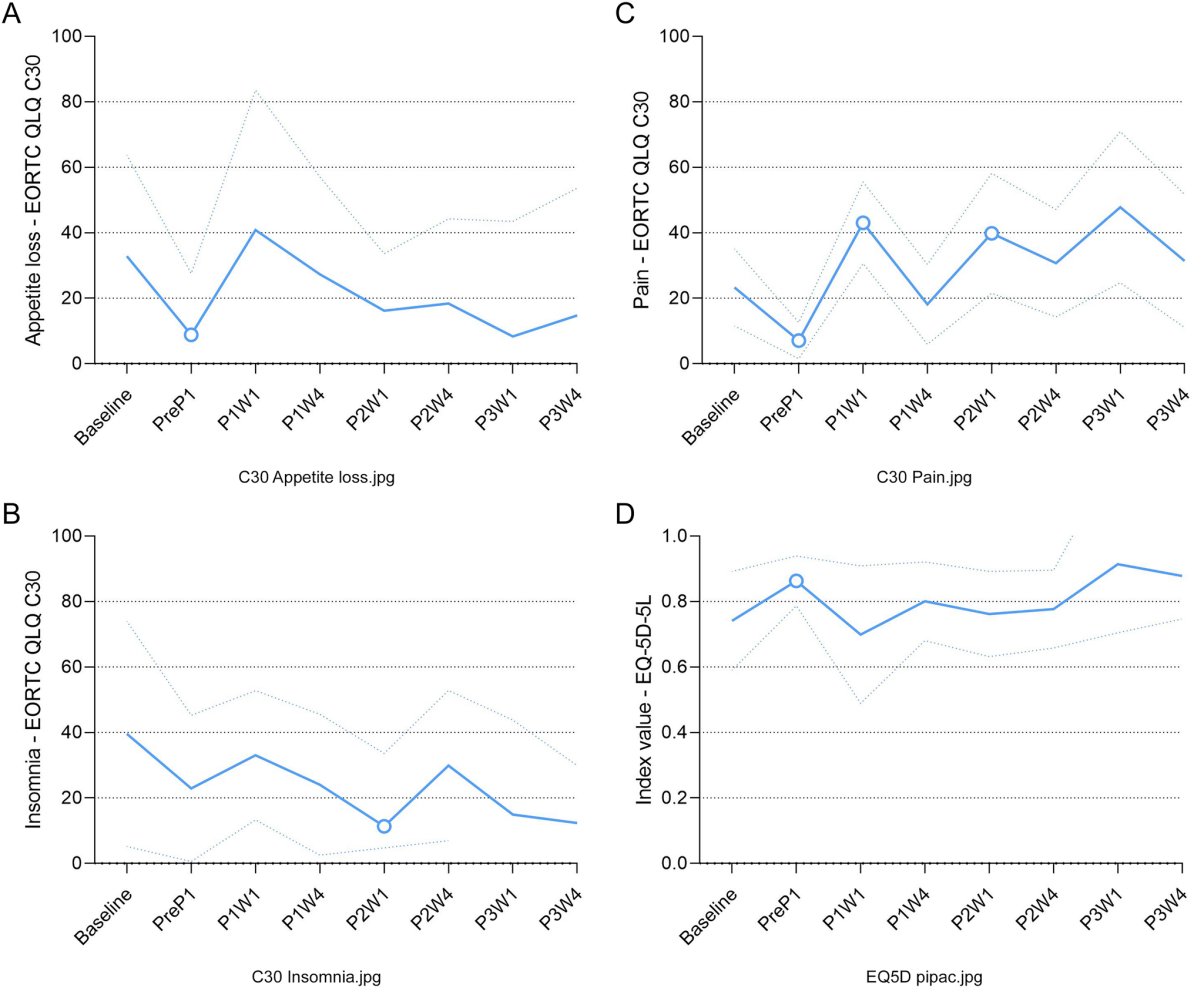
**A**–**D** Symptom scales with a statistically significant mean difference, indicating statistical difference. PreP1 before first PIPAC procedure; P1W1 one week after first PIPAC procedure; P1W4 four weeks after first PIPAC procedure;; P2W1 one week after second PIPAC procedure; P2W4 four weeks after second PIPAC procedure; P3W1 one week after third PIPAC procedure; and P3W4 four weeks after third PIPAC procedure

*Pain*: Compared to baseline, pain significantly decreased after the first cycle of systemic therapy measured one week before the first ePIPAC procedure (MD − 16 [95% CI: − 24 to − 8], *p* < 0.001, CD = 0.90, indicating a major decrease). Subsequently, pain increased one week after the first ePIPAC procedure (MD + 20 [95% CI: 7–33], *p* = 0.003, CD = 0.87, indicating a major increase), and one week after the second ePIPAC procedure (MD + 17 [95% CI: 5–28], *p* = 0.004, CD = 0.92, indicating a major increase) (Fig. 3a).

*Insomnia*: Compared to baseline, insomnia decreased one week after the second ePIPAC procedure (MD − 28 [95% CI: − 47 to − 10], *p* = 0.003, CD = 0.97, indicating a major decrease), at all other time points the score did not deviate from baseline (Fig. 3b).

*Appetite loss*: Compared to baseline, appetite loss decreased after the first cycle of systemic therapy measured one week before the first ePIPAC procedure (MD − 24 [95% CI: − 37 to − 11], *p* = 0.001, CD = 0.94, indicating a major decrease), at all other time points the score did not deviate from baseline (Fig. 3c).

*Abdominal pain*: Compared to baseline, abdominal pain increased one week after the first ePIPAC procedure MD + 16 [95% CI: 5–28], *p* = 0.004, CD = 0.79, indicating a major increase). In addition, compared to baseline, abdominal pain decreased four weeks after the third ePIPAC procedure (MD − 10 [95% CI: − 17–3], *p* = 0.004, CD = 0.40, indicating a minor decrease) (Fig. 3d).

## Discussion

The present study found that 8 of 37 (22%) PROs of patients with unresectable CPM were significantly affected during trial treatment with bidirectional therapy (i.e., palliative systemic therapy and ePIPAC-OX). Deterioration of symptom scales (e.g., overall experienced pain and abdominal pain) but also improvement of both function and symptom scales (e.g., index value, global health status, emotional functioning, C30 summary score, insomnia and appetite loss) was observed during treatment. All deviations from baseline were clinically relevant. Many of the affected PROs improved after the first cycle of systemic therapy. After the first ePIPAC was performed, most PROs did not significantly deviate from baseline anymore. However, overall experienced pain followed a different pattern, as this PROs deteriorated one week after the first and second PIPAC procedure. It should be noted that the deviations were transient and reversible in character and that the 29 other PROs were not affected significantly during trial treatment. This is a promising finding in this palliative patient population undergoing intensive treatment, as worsening of symptoms and functioning is to be expected as disease progresses.

Even though health-related QoL is an important oncological outcome to palliative care interventions, only few studies have investigated PROs of patients undergoing PIPAC monotherapy or PIPAC combined with systemic therapy, both as palliative treatment for peritoneal metastases originating of various primary tumors, as systematically reviewed in 2020 [26]. It was observed that most PROs remained unaffected by PIPAC, while some studies reported an improvement in function scales and some reported an increase in fatigue and pain. This was also found in the CRC-PIPAC-I study that we previously conducted, focusing specifically on ePIPAC-OX as palliative monotherapy for CPM. In this trial, reversible deterioration of several PROs after ePIPAC-OX was reported [27]. Markedly, abdominal pain deteriorated after each PIPAC. When compared to conventional surgery, ePIPAC-OX monotherapy resulted in significantly more abdominal pain [28].

In contrast to the CRC-PIPAC-I study, the present study using intensified bidirectional therapy found transient improvements in six, as well as reversible deteriorations of two PROs during trial treatment. Similar to the CRC-PIPAC study, the present study observed deterioration of pain and abdominal pain after the repetitive ePIPAC procedures.

Various studies in patients with metastatic colorectal cancer have reported a gradual deterioration of PROs during treatment with palliative systemic therapy [29, 30]. In this study, improvements of PROs mainly occurred after the first cycle of systemic therapy. Therefore, it could be reasoned that systemic therapy might have alleviated previously untreated symptoms and thus resulted in a direct first improvement of different PROs. This may for instance be caused by reduction of ascites, thereby relieving of abdominal pressure and improving appetite.

It is also possible that a phenomenon called response shift has occurred. Response shift is defined as a change in one’s perception of quality of life. This means that changes in PROs over the course of a treatment may be either due to actual changes in quality of life or due to changes in one’s perception of quality of life [31]. Response shift can also mitigate the alterations in PROs, thus making it more difficult to detect statistically significant differences. However, this further emphasizes the importance of significant alterations that were observed in the present study.

While ‘abdominal pain’ was one of the most markedly affected PROs in the trial with ePIPAC-OX monotherapy, it was less markedly affected during the present study. In this study, the PRO ‘pain’ seemed to be more strongly affected. The severity of ‘pain’ that was reported in the present study significantly increased at one week after the first and second PIPAC procedure yet went back to baseline level four weeks after the third PIPAC procedure. These results are somewhat comforting, as pain does not seem to increase cumulatively if patients repetitively undergo bidirectional treatment. Simultaneously, these findings underline the importance of adequate post-operative pain management protocols, as pain increased significantly during trial treatment.

Interestingly, a recent Danish trial also observed a significant increase in pain after the third PIPAC [32]. This in addition to other PIPAC-studies have reported the increase in ‘pain,’ which seems to be more extreme in oxaliplatin-based PIPAC than in PIPAC with different oncological agents [33, 34]. The Danish trial reported an increase in pain after the third PIP, whereas we only found significant increases after the first and second PIPAC procedures. This difference might stem from differences in study design: single origin vs multiple origin, Bidirectional therapy vs PIPAC monotherapy, and difference in timing of the QOL questionnaires. However, it might also reflect on the longevity of the PIPAC-induced ‘pain.’ The Danish study conducted the questionnaires at two weeks after a third PIPAC procedure, possibly before the post-operative pain had decreased, whereas we conducted questionnaires one and four weeks after each ePIPAC procedure. Nonetheless, the occurrence of pain in a palliative setting should warrant the implementation of adequate post-operative pain management protocols and patient counseling.

Despite being the first study that prospectively investigated the effect of bidirectional therapy on PROs reported by patients with isolated unresectable CPM, this study has some limitations. The main limitation concerned the small sample size, whereas a larger sample size might have enabled the detection of smaller statistical, but clinically relevant alterations in PROs. Moreover, as the treatment progressed more patients discontinued trial treatment, decreasing the power even further, despite a high response rate to the questionnaires. Therefore, mean differences between baseline and later time points are less likely to be statistically significant, even if they were larger than mean differences of previous time points and despite seeming highly relevant. Furthermore, the extensiveness of the questionnaires might have contributed to a diminishing response rate over time.

In addition, patients were not allowed to have undergone palliative treatment for their CPM before inclusion. As a result, we may have selected relatively fit patients, who are more likely to withstand the negative effects of bidirectional therapy and thus have reported higher function scores and lower symptom scores than less fit patients thereby limiting the generalizability of our results somewhat.

Patient-reported outcomes are of vital importance in oncological research because they provide valuable insights into the patient's perspective, including their quality of life, symptoms, and experienced treatment burden. Therefore, results of the CRC-PIPAC-II trial may be used to design future trials regarding PIPAC, with or without systemic therapy, as palliative treatment option for patients with isolated unresectable CPM. The addition of ePIPAC to systemic therapy appears to be reasonably well tolerated. However, physicians should take treatment-related pain into account when counseling patients and might consider implementing more intense post-operative pain management protocols, or investigate a PIPAC regimen that is less likely to cause pain.

## Conclusion

Patients with isolated unresectable CPM treated with first-line palliative systemic therapy and oxaliplatin-based ePIPAC showed statistically significant and clinically relevant alterations of different PROs during trial treatment, which concerned both improvements and deteriorations. All deteriorated PROs eventually returned to baseline scores after finishing trial treatment. However, adequate post-operative pain management protocols and preoperative counseling are warranted. As most PROs remained unaffected during trial treatment and some even improved, bidirectional therapy seems to be reasonably well tolerated, based on PRO exploration. These findings can be used to inform patients about the burden and chances offered by the bidirectional therapy. In addition, they may help in the design in future randomized clinical trials regarding palliative bidirectional therapy with PIPAC.

## Supplementary Information

Below is the link to the electronic supplementary material.

Supplementary file1 (DOCX 59 KB)
