# Supplementary material for: Patient-reported outcomes during first-line palliative systemic therapy alternated with pressurized intraperitoneal aerosol chemotherapy for unresectable colorectal peritoneal metastases: a single-arm phase II trial (CRC-PIPAC-II)
Source: Surg Endosc. 2024 Sep 16;38(11):6566–76. doi: 10.1007/s00464-024-11185-z (PMC11525311; doi:10.1007/s00464-024-11185-z)
Supplement: Supplementary file 1 — Supplementary file1 (DOCX 59 KB) [file 464_2024_11185_MOESM1_ESM.docx]

| **Supplementary tabel 1A L**inear mixed modeling analyses of function scales, with comparisons between baseline and subsequent timepoints. | | | | |
| --- | --- | --- | --- | --- |
| **Function scales** | **Mean difference** | **95% CI** | **p-value** | **Cohen's d** |
| **Index value (EQ-5D-5L)** |  |  |  |  |
| baseline vs. prior to first procedure | +0.12 | 0.05 – 0.19 | 0.001 | 1.02 |
| baseline vs. one week after first procedure | -0.04 | -0.17 – 0.08 | 0.51 | - |
| baseline vs. four weeks after first procedure | +0.06 | -0.02 – 0.14 | 0.15 | - |
| baseline vs. one week after second procedure | +0.02 | -0.04 – 0.08 | 0.53 | - |
| baseline vs. four weeks after second procedure | +0.04 | -0.02 – 0.09 | 0.22 | - |
| baseline vs. one week after third procedure | +0.17 | -0.03 – 0.37 | 0.093 | - |
| baseline vs. four weeks after third procedure | +0.14 | -0.001 – 0.27 | 0.051 | - |
| **Visual analogue scale (EQ-5D-5L)** |  |  |  |  |
| baseline vs. prior to first procedure | +5 | -2 – 12 | 0.20 | - |
| baseline vs. one week after first procedure | -2 | -13 – 8 | 0.65 | - |
| baseline vs. four weeks after first procedure | +4 | -4 – 12 | 0.38 | - |
| baseline vs. one week after second procedure | -2 | -8 – 5 | 0.66 | - |
| baseline vs. four weeks after second procedure | +4 | -3 – 10 | 0.23 | - |
| baseline vs. one week after third procedure | +9 | -5 – 22 | 0.20 | - |
| baseline vs. four weeks after third procedure | +5 | -6 – 16 | 0.36 | - |
| **Global Health status (EORTC-QLQ-C30)** |  |  |  |  |
| baseline vs. prior to first procedure | +13 | 5 – 21 | 0.001 | 0.69 |
| baseline vs. one week after first procedure | -5 | -22 – 11 | 0.51 | - |
| baseline vs. four weeks after first procedure | +2 | -9 – 14 | 0.69 | - |
| baseline vs. one week after second procedure | -6 | -16 – 5 | 0.29 | - |
| baseline vs. four weeks after second procedure | 0 | -10 – 11 | 0.96 | - |
| baseline vs. one week after third procedure | -11 | -26 – 3 | 0.13 | - |
| baseline vs. four weeks after third procedure | +12 | 6 – 18 | <0.001 | 0.62 |
| **Physical functioning (EORTC-QLQ-C30)** |  |  |  |  |
| baseline vs. prior to first procedure | +7 | -6 – 19 | 0.28 | - |
| baseline vs. one week after first procedure | -11 | -25 – 3 | 0.14 | - |
| baseline vs. four weeks after first procedure | +4 | -6 – 15 | 0.40 | - |
| baseline vs. one week after second procedure | -4 | -13 – 5 | 0.39 | - |
| baseline vs. four weeks after second procedure | 0 | -6 – 7 | 0.91 | - |
| baseline vs. one week after third procedure | -11 | -43 – 21 | 0.51 | - |
| baseline vs. four weeks after third procedure | +3 | -8 – 15 | 0.60 | - |
| **Role functioning (EORTC-QLQ-C30)** |  |  |  |  |
| baseline vs. prior to first procedure | +16 | +2 – 30 | 0.03 | - |
| baseline vs. one week after first procedure | -9 | -26 – 9 | 0.33 | - |
| baseline vs. four weeks after first procedure | +10 | -7 – 26 | 0.27 | - |
| baseline vs. one week after second procedure | -15 | -30 – 1 | 0.07 | - |
| baseline vs. four weeks after second procedure | +2 | -14 – 17 | 0.84 | - |
| baseline vs. one week after third procedure | +7 | -14 – 27 | 0.49 | - |
| baseline vs. four weeks after third procedure | +1 | -18 – 21 | 0.91 | - |
|  |  |  |  |  |
| **Supplementary tabel 1A L**inear mixed modeling analyses of function scales, with comparisons between baseline and subsequent timepoints. (continued) | | | | |
| **Function scales** | **Mean difference** | **95% CI** | **p-value** | **Cohen's d** |
| **Emotional functioning (EORTC-QLQ-C30)** |  |  |  |  |
| baseline vs. prior to first procedure | +13 | 4 – 23 | 0.008 | - |
| baseline vs. one week after first procedure | +6 | -3 – 16 | 0.18 | - |
| baseline vs. four weeks after first procedure | +7 | -1 – 15 | 0.10 | - |
| baseline vs. one week after second procedure | +10 | 1 – 19 | 0.03 | - |
| baseline vs. four weeks after second procedure | +7 | -5 – 18 | 0.26 | - |
| baseline vs. one week after third procedure | +12 | -2 – 27 | 0.10 | - |
| baseline vs. four weeks after third procedure | +20 | 11 – 29 | <0.001 | 1.07 |
| **Cognitive functioning (EORTC-QLQ-C30)** |  |  |  |  |
| baseline vs. prior to first procedure | +6 | -4 – 17 | 0.24 | - |
| baseline vs. one week after first procedure | 0 | -11 – 11 | 0.98 | - |
| baseline vs. four weeks after first procedure | +8 | -2 – 18 | 0.10 | - |
| baseline vs. one week after second procedure | +5 | -6 – 17 | 0.37 | - |
| baseline vs. four weeks after second procedure | -4 | -20 – 12 | 0.62 | - |
| baseline vs. one week after third procedure | -6 | -22 – 10 | 0.49 | - |
| baseline vs. four weeks after third procedure | -1 | -9 – 6 | 0.71 | - |
| **Social functioning (EORTC-QLQ-C30)** |  |  |  |  |
| baseline vs. prior to first procedure | +17 | 3 – 31 | 0.02 | - |
| baseline vs. one week after first procedure | +1 | -17 – 19 | 0.86 | - |
| baseline vs. four weeks after first procedure | +8 | -10 – 26 | 0.46 | - |
| baseline vs. one week after second procedure | -2 | -19 – 14 | 0.73 | - |
| baseline vs. four weeks after second procedure | +13 | -1 – 27 | 0.08 | - |
| baseline vs. one week after third procedure | -3 | -23 – 16 | 0.78 | - |
| baseline vs. four weeks after third procedure | +3 | -19 – 25 | 0.71 | - |
| **C30 summary score (EORTC-QLQ-C30)** |  |  |  |  |
| baseline vs. prior to first procedure | +12 | 6 – 18 | <0.001 | 0.93 |
| baseline vs. one week after first procedure | -7 | -16 – 3 | -.16 | - |
| baseline vs. four weeks after first procedure | +1 | -7 – 9 | 0.82 | - |
| baseline vs. one week after second procedure | -3 | -10 – 3 | 0.26 | - |
| baseline vs. four weeks after second procedure | -1 | -9 – 7 | 0.77 | - |
| baseline vs. one week after third procedure | -7 | -16 – 1 | 0.10 | - |
| baseline vs. four weeks after third procedure | -2 | -10 – 6 | 0.65 | - |
| **Anxiety (EORTC-QLQ-CR29)** |  |  |  |  |
| baseline vs. prior to first procedure | +5 | 3 – 23 | 0.009 | - |
| baseline vs. one week after first procedure | +5 | 0 – 18 | 0.05 | - |
| baseline vs. four weeks after first procedure | +6 | -9 – 16 | 0.53 | - |
| baseline vs. one week after second procedure | +8 | -27 – 8 | 0.23 | - |
| baseline vs. four weeks after second procedure | +5 | -3 – 17 | 0.17 | - |
| baseline vs. one week after third procedure | +6 | -12 – 12 | 0.99 | - |
| baseline vs. four weeks after third procedure | 0 | 0 – 0 | 0.12 | - |

| **Supplementary tabel 1A L**inear mixed modeling analyses of function scales, with comparisons between baseline and subsequent timepoints. (continued) | | | | |
| --- | --- | --- | --- | --- |
| **Function scales** | **Mean difference** | **95% CI** | **p-value** | **Cohen's d** |
| **Weight (EORTC-QLQ-CR29)** |  |  |  |  |
| baseline vs. prior to first procedure | +4 | -12 – 21 | 0.60 | - |
| baseline vs. one week after first procedure | +11 | -7 – 28 | 0.24 | - |
| baseline vs. four weeks after first procedure | +3 | -15 – 21 | 0.74 | - |
| baseline vs. one week after second procedure | -7 | -26 – 12 | 0.45 | - |
| baseline vs. four weeks after second procedure | -6 | -24 – 13 | 0.56 | - |
| baseline vs. one week after third procedure | 0 | -21 – 21 | 0.99 | - |
| baseline vs. four weeks after third procedure | -4 | -24 – 17 | 0.74 | - |
| **Body image (EORTC-QLQ-CR29)** |  |  |  |  |
| baseline vs. prior to first procedure | -5 | -13 – 2 | 0.17 | - |
| baseline vs. one week after first procedure | -3 | -11 - 5 | 0.42 | - |
| baseline vs. four weeks after first procedure | -6 | -11 – 0 | 0.06 | - |
| baseline vs. one week after second procedure | +5 | -2 – 12 | 0.13 | - |
| baseline vs. four weeks after second procedure | -5 | -17 – 7 | 0.46 | - |
| baseline vs. one week after third procedure | -2 | -12 – 7 | 0.59 | - |
| baseline vs. four weeks after third procedure | +1 | -5 - 8 | 0.73 | - |
| *CI* Confidence interval; *PRO* patient reported outcome  **Supplementary tabel 1B L**inear mixed modeling analyses of symptom scales, with comparisons between baseline and subsequent timepoints. | | | | |
| **Symptom scales** | **Mean difference** | **95% CI** | **p-value** | **Cohen's d** |
| **Fatigue (EORTC-QLQ-C30)** |  |  |  |  |
| baseline vs. prior to first procedure | -8 | -21 – 4 | 0.19 | - |
| baseline vs. one week after first procedure | +13 | -4 – 30 | 0.13 | - |
| baseline vs. four weeks after first procedure | +5 | -12 – 23 | 0.56 | - |
| baseline vs. one week after second procedure | +20 | -2 – 41 | 0.07 | - |
| baseline vs. four weeks after second procedure | -1 | -16 – 13 | 0.84 | - |
| baseline vs. one week after third procedure | +12 | -20 – 44 | 0.37 | - |
| baseline vs. four weeks after third procedure | -8 | -40 – 24 | 0.55 | - |
| **Nausea/vomiting (EORTC-QLQ-C30)** |  |  |  |  |
| baseline vs. prior to first procedure | -12 | -24 – 0 | 0.05 | - |
| baseline vs. one week after first procedure | +12 | -10 – 34 | 0.30 | - |
| baseline vs. four weeks after first procedure | +1 | -14 – 16 | 0.90 | - |
| baseline vs. one week after second procedure | +10 | -8 – 28 | 0.29 | - |
| baseline vs. four weeks after second procedure | -5 | -22 – 13 | 0.62 | - |
| baseline vs. one week after third procedure | +22 | 0 – 43 | 0.05 | - |
| baseline vs. four weeks after third procedure | -6 | -19 – 8 | 0.43 | - |
|  |  |  |  |  |

| **Supplementary tabel 1B L**inear mixed modeling analyses of symptom scales, with comparisons between baseline and subsequent timepoints. (continued) | | | | |
| --- | --- | --- | --- | --- |
| **Symptom scales** | **Mean difference** | **95% CI** | **p-value** | **Cohen’s d** |
| **Pain (EORTC-QLQ-C30)** |  |  |  |  |
| baseline vs. prior to first procedure | -16 | -24 – -8 | <0.001 | 0.90 |
| baseline vs. one week after first procedure | +20 | 7 – 33 | 0.003 | 0.87 |
| baseline vs. four weeks after first procedure | -5 | -18 – 8 | 0.41 | - |
| baseline vs. one week after second procedure | +17 | 5 – 28 | 0.004 | 0.63 |
| baseline vs. four weeks after second procedure | +7 | -4 – 19 | 0.20 | - |
| baseline vs. one week after third procedure | +24 | 4 – 45 | 0.02 | - |
| baseline vs. four weeks after third procedure | +5 | -3 – 19 | 0.15 | - |
| **Dyspnea (EORTC-QLQ-C30)** |  |  |  |  |
| baseline vs. prior to first procedure | +2 | -7 – 10 | 0.70 | - |
| baseline vs. one week after first procedure | +4 | -5 – 14 | 0.36 | - |
| baseline vs. four weeks after first procedure | 0 | -8 – 7 | 0.93 | - |
| baseline vs. one week after second procedure | +6 | -6 – 18 | 0.33 | - |
| baseline vs. four weeks after second procedure | +4 | -6 – 15 | 0.41 | - |
| baseline vs. one week after third procedure | +5 | -9 – 20 | 0.48 | - |
| baseline vs. four weeks after third procedure | +2 | -7 – 10 | 0.67 | - |
| **Insomnia (EORTC-QLQ-C30)** |  |  |  |  |
| baseline vs. prior to first procedure | -17 | -31 – -3 | 0.02 | - |
| baseline vs. one week after first procedure | -7 | -28 – 15 | 0.55 | - |
| baseline vs. four weeks after first procedure | -16 | -37 – 6 | 0.16 | - |
| baseline vs. one week after second procedure | -28 | -47 – -10 | 0.003 | 0.003 |
| baseline vs. four weeks after second procedure | -10 | -29 – 9 | 0.32 | - |
| baseline vs. one week after third procedure | -25 | -48 – -1 | 0.04 | - |
| baseline vs. four weeks after third procedure | -27 | -48 – -6 | 0.01 | - |
| **Appetite loss (EORTC-QLQ-C30)** |  |  |  |  |
| baseline vs. prior to first procedure | -24 | -37 – -11 | 0.001 | 0.93 |
| baseline vs. one week after first procedure | 8 | -17 – 33 | 0.52 | - |
| baseline vs. four weeks after first procedure | -6 | -28 – 17 | 0.63 | - |
| baseline vs. one week after second procedure | -17 | -35 – 2 | 0.07 | - |
| baseline vs. four weeks after second procedure | -14 | -34 – 5 | 0.14 | - |
| baseline vs. one week after third procedure | -25 | -58 – 9 | 0.15 | - |
| baseline vs. four weeks after third procedure | -18 | -55 – 19 | 0.34 | - |
| **Constipation (EORTC-QLQ-C30)** |  |  |  |  |
| baseline vs. prior to first procedure | -7 | -7 – 21 | 0.35 | - |
| baseline vs. one week after first procedure | +4 | -19 – 11 | 0.59 | - |
| baseline vs. four weeks after first procedure | -2 | -13 – 17 | 0.81 | - |
| baseline vs. one week after second procedure | +3 | -19 – 13 | 0.72 | - |
| baseline vs. four weeks after second procedure | +2 | -17 – 14 | 0.83 | - |
| baseline vs. one week after third procedure | -3 | -15 – 20 | 0.77 | - |
| baseline vs. four weeks after third procedure | n.a. | n.a. | n.a. |  |
| **Supplementary tabel 1B L**inear mixed modeling analyses of symptom scales, with comparisons between baseline and subsequent timepoints. (continued) | | | | |
| **Symptom scales (continued)** | **Mean difference** | **95% CI** | **p-value** | **Cohen's d** |
| **Diarrhea (EORTC-QLQ-C30)** |  |  |  |  |
| baseline vs. prior to first procedure | -1 | -16 – 13 | 0.86 | - |
| baseline vs. one week after first procedure | +19 | -6 – 44 | 0.14 | - |
| baseline vs. four weeks after first procedure | +3 | -19 – 25 | 0.79 | - |
| baseline vs. one week after second procedure | 0 | -13 – 13 | 0.99 | - |
| baseline vs. four weeks after second procedure | +11 | -19 – 41 | 0.33 | - |
| baseline vs. one week after third procedure | +5 | -17 – 27 | 0.63 | - |
| baseline vs. four weeks after third procedure | +1 | -15 – 16 | 0.91 | - |
| **Financial issues (EORTC-QLQ-C30)** |  |  |  |  |
| baseline vs. prior to first procedure | -1 | -12 – 13 | 0.92 | - |
| baseline vs. one week after first procedure | -1 | -12 – 15 | 0.83 | - |
| baseline vs. four weeks after first procedure | +1 | -15 – 12 | 0.86 | - |
| baseline vs. one week after second procedure | 0 | -14 – 15 | 0.96 | - |
| baseline vs. four weeks after second procedure | -1 | -13 – 15 | 0.92 | - |
| baseline vs. one week after third procedure | +2 | -17 – 14 | 0.85 | - |
| baseline vs. four weeks after third procedure | -2 | -13 – 18 | 0.78 | - |
| **Urinary frequency (EORTC-QLQ-CR29)** |  |  |  |  |
| baseline vs. prior to first procedure | -8 | -19 – 3 | 0.13 | - |
| baseline vs. one week after first procedure | -5 | -13 – 3 | 0.18 | - |
| baseline vs. four weeks after first procedure | -13 | -26 – 5 | 0.18 | - |
| baseline vs. one week after second procedure | -7 | -17 – 4 | 0.22 | - |
| baseline vs. four weeks after second procedure | -8 | -20 – 2 | 0.12 | - |
| baseline vs. one week after third procedure | -7 | -28 - 9 | 0.32 | - |
| baseline vs. four weeks after third procedure | -9 | -21 – 4 | 0.16 | - |
| **Urinary incontinence (EORTC-QLQ-CR29)** |  |  |  |  |
| baseline vs. prior to first procedure | -3 | -10 – 3 | 0.30 | - |
| baseline vs. one week after first procedure | -3 | -10 – 3 | 0.33 | - |
| baseline vs. four weeks after first procedure | -3 | -10 – 1 | 0.34 | - |
| baseline vs. one week after second procedure | -3 | -11 – 4 | 0.36 | - |
| baseline vs. four weeks after second procedure | -1 | -8 – 6 | 0.83 | - |
| baseline vs. one week after third procedure | 0 | -8 – 8 | 0.93 | - |
| baseline vs. four weeks after third procedure | 4 | -12 – 4 | 0.31 | - |
| **Dysuria (EORTC-QLQ-CR29)** |  |  |  |  |
| baseline vs. prior to first procedure | 0 | -5 – 5 | 0.97 | - |
| baseline vs. one week after first procedure | -2 | -5 – 2 | 0.30 | - |
| baseline vs. four weeks after first procedure | -2 | -5 – 2 | 0.30 | - |
| baseline vs. one week after second procedure | 0 | 0 – 0 | 1.0 | - |
| baseline vs. four weeks after second procedure | -2 | -5 – 2 | 0.30 | - |
| baseline vs. one week after third procedure | -3 | -8 – 1 | 0.98 | - |
| baseline vs. four weeks after third procedure | -1 | -3 – 1 | 0.35 | - |

| **Supplementary tabel 1B L**inear mixed modeling analyses of symptom scales, with comparisons between baseline and subsequent timepoints. (continued) | | | | |
| --- | --- | --- | --- | --- |
| **Symptom scales** | **Mean difference** | **95% CI** | **p-value** | **Cohen’s d** |
| **Abdominal pain (EORTC-QLQ-CR29)** |  |  |  |  |
| baseline vs. prior to first procedure | -14 | -25 – -4 | 0.009 | 0.65 |
| baseline vs. one week after first procedure | 16 | 5 – 28 | 0.004 | 0.79 |
| baseline vs. four weeks after first procedure | -6 | -22 – 1- | 0.49 | - |
| baseline vs. one week after second procedure | 9 | -8 – 26 | 0.31 | - |
| baseline vs. four weeks after second procedure | -3 | -15 – 10 | 0.66 | - |
| baseline vs. one week after third procedure | 6 | -9 – 22 | 0.27 | - |
| baseline vs. four weeks after third procedure | -10 | -17 – 3 | 0.004 | 0.40 |
| **Buttock pain (EORTC-QLQ-CR29)** |  |  |  |  |
| baseline vs. prior to first procedure | -2 | -7 – 3 | 0.96 | - |
| baseline vs. one week after first procedure | +3 | -8 – 2 | 0.29 | - |
| baseline vs. four weeks after first procedure | -2 | -7 – 4 | 0.53 | - |
| baseline vs. one week after second procedure | -2 | -7 – 4 | 0.55 | - |
| baseline vs. four weeks after second procedure | +1 | -6 – 4 | 0.74 | - |
| baseline vs. one week after third procedure | +2 | -8 – 4 | 0.51 | - |
| baseline vs. four weeks after third procedure | +2 | -8 – 4 | 0.51 | - |
| **bloating (EORTC-QLQ-CR29)** |  |  |  |  |
| baseline vs. prior to first procedure | -12 | -25 – 2 | 0.09 | - |
| baseline vs. one week after first procedure | +12 | -3 – 27 | 0.11 | - |
| baseline vs. four weeks after first procedure | +1 | -13 – 16 | 0.84 | - |
| baseline vs. one week after second procedure | +13 | -4 – 30 | 0.15 | - |
| baseline vs. four weeks after second procedure | +5 | -7 – 18 | 0.42 | - |
| baseline vs. one week after third procedure | +23 | 3 – 42 | 0.03 | - |
| baseline vs. four weeks after third procedure | -1 | -1 – 0 | 0.04 | - |
| **Blood in stool (EORTC-QLQ-CR29)** |  |  |  |  |
| baseline vs. prior to first procedure | +6 | -4 – 17 | 0.23 | - |
| baseline vs. one week after first procedure | +3 | -11 – 18 | 0.65 | - |
| baseline vs. four weeks after first procedure | +4 | -8 – 16 | 0.46 | - |
| baseline vs. one week after second procedure | 0 | -9 – 9 | 1.00 | - |
| baseline vs. four weeks after second procedure | +4 | -6 – 15 | 0.36 | - |
| baseline vs. one week after third procedure | +3 | -13 – 19 | 0.69 | - |
| baseline vs. four weeks after third procedure | 0 | -9 – 9 | 1.00 | - |
| **Dry mouth (EORTC-QLQ-CR29)** |  |  |  |  |
| baseline vs. prior to first procedure | -4 | -23 – 11 | 0.49 | - |
| baseline vs. one week after first procedure | -2 | -23 – 14 | 0.63 | - |
| baseline vs. four weeks after first procedure | 5 | -21 – 17 | 0.83 | - |
| baseline vs. one week after second procedure | 1 | -25 – 15 | 0.61 | - |
| baseline vs. four weeks after second procedure | 13 | -20 – 19 | 0.96 | - |
| baseline vs. one week after third procedure | 6 | -35 – 8 | 0.22 | - |
| baseline vs. four weeks after third procedure | -20 | -27 – 16 | 0.59 | - |
|  |  |  |  |  |
| **Supplementary tabel 1B L**inear miyxed modeling analyses of symptom scales, with comparisons between baseline and subsequent timepoints. (continued) | | | | |
| **Symptom scales** | **Mean difference** | **95% CI** | **p-value** | **Cohen's d** |
| **Hairloss (EORTC-QLQ-CR29)** |  |  |  |  |
| baseline vs. prior to first procedure | +1 | -17 – 18 | 0.95 | - |
| baseline vs. one week after first procedure | +8 | -11 – 27 | 0.39 | - |
| baseline vs. four weeks after first procedure | +12 | -7 – 31 | 0.22 | - |
| baseline vs. one week after second procedure | +11 | -10 – 31 | 0.20 | - |
| baseline vs. four weeks after second procedure | +14 | -6 – 34 | 0.16 | - |
| baseline vs. one week after third procedure | -1 | -22 – 23 | 0.96 | - |
| baseline vs. four weeks after third procedure | -1 | -22 - 23 | 0.96 | - |
| **Taste (EORTC-QLQ-CR29)** |  |  |  |  |
| baseline vs. prior to first procedure | -13 | -26 – 0 | 0.06 | - |
| baseline vs. one week after first procedure | +7 | -6 – 19 | 0.29 | - |
| baseline vs. four weeks after first procedure | -7 | -19 – 4 | 0.20 | - |
| baseline vs. one week after second procedure | -1 | -16 – 14 | 0.87 | - |
| baseline vs. four weeks after second procedure | +4 | -12 – 20 | 0.61 | - |
| baseline vs. one week after third procedure | +7 | -3 – 17 | 0.18 | - |
| baseline vs. four weeks after third procedure | 0 | -1 – 1 | 0.78 | - |
| **Flatulence (EORTC-QLQ-CR29)** |  |  |  |  |
| baseline vs. prior to first procedure | +3 | -1 – 8 | 0.11 | - |
| baseline vs. one week after first procedure | +2 | -4 – 8 | 0.53 | - |
| baseline vs. four weeks after first procedure | +2 | -4 – 9 | 0.52 | - |
| baseline vs. one week after second procedure | +7 | -5 – 18 | 0.28 | - |
| baseline vs. four weeks after second procedure | +5 | -2 – 12 | 0.14 | - |
| baseline vs. one week after third procedure | +2 | -4 – 9 | 0.54 | - |
| baseline vs. four weeks after third procedure | +2 | 0 – 4 | 0.13 | - |
| **Fecal incontinence (EORTC-QLQ-CR29)** |  |  |  |  |
| baseline vs. prior to first procedure | 0 | -5 – 5 | 0.99 | - |
| baseline vs. one week after first procedure | -3 | -10 – 4 | 0.38 | - |
| baseline vs. four weeks after first procedure | -3 | -7 – 1 | 0.15 | - |
| baseline vs. one week after second procedure | -1 | -37 – 36 | 0.91 | - |
| baseline vs. four weeks after second procedure | +1 | -4 – 6 | 0.67 | - |
| baseline vs. one week after third procedure | +4 | -17 – 18 | 0.46 | - |
| baseline vs. four weeks after third procedure | -3 | -8 – 0 | 0.62 | - |
| **Sore skin (EORTC-QLQ-CR29)** |  |  |  |  |
| baseline vs. prior to first procedure | +4 | -6 – 13 | 0.43 | - |
| baseline vs. one week after first procedure | +5 | -6 – 15 | 0.38 | - |
| baseline vs. four weeks after first procedure | 0 | -6 – 5 | 0.89 | - |
| baseline vs. one week after second procedure | -2 | -2 – 6 | 0.29 | - |
| baseline vs. four weeks after second procedure | +11 | 0 – 21 | 0.04 | - |
| baseline vs. one week after third procedure | +3 | -4 – 11 | 0.36 | - |
| baseline vs. four weeks after third procedure | -1 | -4 – 1 | 0.34 | - |

| **Supplementary tabel 1B L**inear miyxed modeling analyses of symptom scales, with comparisons between baseline and subsequent timepoints. (continued) | | | | |
| --- | --- | --- | --- | --- |
| **Symptom scales** | **Mean difference** | **95% CI** | **p-value** | **Cohen's d** |
| **Stool frequency (EORTC-QLQ-CR29)** |  |  |  |  |
| baseline vs. prior to first procedure | -7 | -15 – 1 | 0.07 | - |
| baseline vs. one week after first procedure | +4 | -7 – 15 | 0.45 | - |
| baseline vs. four weeks after first procedure | -10 | -20 – 1 | 0.07 | - |
| baseline vs. one week after second procedure | -4 | -20 – 11 | 0.58 | - |
| baseline vs. four weeks after second procedure | -7 | -17 – 2 | 0.13 | - |
| baseline vs. one week after third procedure | -7 | -22 – 7 | 0.30 | - |
| baseline vs. four weeks after third procedure | -14 | -26 – -3 | 0.01 | - |
| **Embarrassment (EORTC-QLQ-CR29)** |  |  |  |  |
| baseline vs. prior to first procedure | +3 | -3 – 8 | 0.99 | - |
| baseline vs. one week after first procedure | +12 | -2 – 25 | 0.08 | - |
| baseline vs. four weeks after first procedure | -2 | -5 – 2 | 0.35 | - |
| baseline vs. one week after second procedure | +5 | -6 – 16 | 0.39 | - |
| baseline vs. four weeks after second procedure | +7 | -5 – 19 | 0.26 | - |
| baseline vs. one week after third procedure | +5 | -6 – 16 | 0.39 | - |
| baseline vs. four weeks after third procedure | 0 | 0 – 0 | 0.85 | - |
| **Stoma care problems (EORTC-QLQ-CR29)** |  |  |  |  |
| baseline vs. prior to first procedure | -11 | -37 – 59 | 0.75 | - |
| baseline vs. one week after first procedure | -11 | -37 – 59 | 0.75 | - |
| baseline vs. four weeks after first procedure | -11 | -37 – 59 | 0.75 | - |
| baseline vs. one week after second procedure | -11 | -37 – 59 | 0.94 | - |
| baseline vs. four weeks after second procedure | -11 | -37 - 59 | 0.94 | - |
| baseline vs. one week after third procedure | n.a. | n.a. | n.a. | - |
| baseline vs. four weeks after third procedure | n.a. | n.a. | n.a. | - |

*CI* Confidence interval; *PRO* patient reported outcome
